# Hydrophobic covalent organic frameworks: a green synthesis approach for efficient oil/water separation†

**DOI:** 10.1039/d4ra08201j

**Published:** 2025-02-13

**Authors:** Bilian Li, Quanmei Duan, Lishen Yang, Tan Feng, Chang Ru, Xin Zhang, Hui Zhao, Can-Peng Li

**Affiliations:** a School of Chemical Science and Technology, Yunnan University 2 North Cuihu Road Kunming 650091 People's Republic of China lcppp1974@sina.com; b Laboratory for Conservation and Utilization of Bioresource, Yunnan University 2 North Cuihu Road Kunming 650091 People's Republic of China zhaohui@ynu.edu.cn; c Research Center for Safety and Environment (Double Carbon Research Center), Pipe China Institute of Science and Technology Tianjin 300457 China

## Abstract

Rapid economic development has led to oil pollution and energy shortage. Thus, it is highly desirable to develop an efficient and environment-friendly approach for oil/water (O/W) separation. Herein, we report a simple and green method for preparing macroscopic COF AG and AG. COF AG was rapidly synthesized at room temperature, washed and freeze-dried to prepare COF AG without any adhesives or additives. Due to its strong hydrophobicity, COF AG is used as an absorbent for removing organic pollutants in O/W separation, and has a certain demulsification performance, which has a certain application prospect in the field of O/W separation. The hydrophobic COF AG material was combined with MA, which was synthesized in one step at room temperature, avoiding the long reaction conditions of traditional high temperature and high-pressure reaction, as well as the post-modification process and complex washing steps. The superhydrophobic sponge material was rapidly prepared. The introduction of MA reduced the amount of COF monomer, improved the adsorption capacity of the material for organic solvents and oil samples, increased from 37 times of the previous maximum adsorption weight to more than 120 times, and the demulsification capacity of O/W emulsion increased to more than 99%, with the ability of direct separation and continuous separation of O/W. Therefore, the prepared superhydrophobic sponge has high adsorption capacity and good reusability, and can be used for O/W separation. This work not only provides a strategy for the construction of functional COF, but also opens up a way for the growth of COF on different carriers for O/W separation.

## Abstract 2

The rapid economic development has led to oil pollution and energy shortages. In this paper, a hydrophobic covalent organic framework of COF-AG was synthesized rapidly at room temperature, which has high oil–water separation performance.

## Graphical abstract

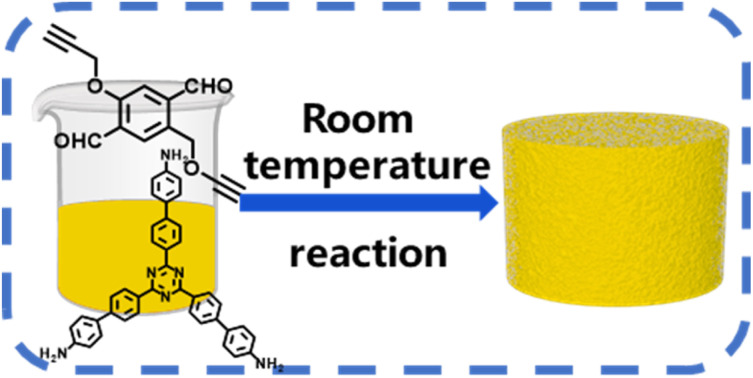

## 1. Introduction

Oil is an important energy source for modern civilized society. The huge supply of petroleum and its by-products has led to the rapid development of petroleum exploration worldwide.^1^ However, once oil or related chemicals are leaked into the environment, huge environmental damage and economic losses will be caused.^2^ Appropriate methods need to be used to collect and separate spilled oil or related chemicals to reduce economic losses and environmental pollution. In the face of oil spill accidents, methods such as *in situ* combustion, biodegradation and physical absorption are often adopted to reduce the negative impact of the accident.^3–5^ Physical adsorption can recover spilled oil and minimize economic losses, so it is favored by people.

Sponges are an excellent oil absorbing material because they have high porosity and can be recovered by compression or centrifugation to release the absorbed liquid. However, as most commercial sponges lack the ability to selectively absorb oil, they cannot be used directly as oil absorbent. Therefore, many researchers try to modify sponges to obtain super hydrophobic sponges. Qiu *et al.*^6^ prepared a superhydrophobic sponge by coating a polyurethane (PU) sponge skeleton with superhydrophobic sepiolite. Xue^7^ prepared a superhydrophobic polydimethylsiloxane/copper/terephthalate/PU sponge. Zheng^8^ polyphenol amino modification method is used to step in the preparation of super hydrophobic melamine (MA) sponge. Li^9^ coated MA sponge with Fe_3_O_4_ nanoparticles modified with fluoroalkyl silane by drip coating to make the sponge have magnetic and superhydrophobic properties. However, most of the above methods require high temperature and complex synthesis steps.

COF are crystalline organic materials with a wide range of applications due to their porosity, adjustable structure and precise chemical properties. In recent years, COF, as an emerging adsorbent, have shown better performance in many adsorption and separation fields.^10–12^ However, COF are usually generated in the form of powder, which is not easy to collect in the field of adsorption application has become an obstacle to their development. In order to make COF play their own advantages in the field of adsorption, synthesis of macroscopic COF is particularly important. COF aerogel (COF AG), as the most important member of macroscopic COF, combines the performance of COF with the advantages of AG and becomes a hot topic in current research.^13^

Porous polymer nanocomposites, which typically consist of a polymer matrix reinforced with inorganic nanoparticles. While these composites exhibit enhanced mechanical properties and thermal stability, their porosity and crystalline nature are often limited, making them less suitable for applications requiring precise pore size control and high surface areas.^14^ Metal–Organic Frameworks (MOFs), are known for their exceptional porosity and large surface areas. These materials are formed through the coordination of metal ions with organic ligands, resulting in highly ordered crystalline structures. However, the stability of MOFs under harsh conditions, such as high temperatures or in the presence of certain solvents, remains a challenge.^15^ Polymeric Organic Frameworks (POPs), similar to COFs, are constructed through covalent bonds between organic molecules. However, POPs often lack the long-range order and crystalline structure that are characteristic of COFs. This lack of crystallinity can lead to reduced porosity and surface area, limiting their potential applications.^16^ In contrast, COF materials exhibit enhanced stability due to their covalent bonding, which makes them more resilient to degradation and offer a high degree of crystallinity and pore size control, making them more versatile for a range of applications.

As mentioned above, COF AG mainly includes compound COF AG. Composite COF AG is based on chitosan AG^17^ or reduced graphene oxide AG^18^ to form a COF layer externally, while pure COF AG as a whole is a kind of material formed by COF themselves,^13,19^ without other AG as a base. In order to avoid the overall structural shrinkage caused by the capillary tension in the gel pore, the drying process of pure COF AG was mainly carried out by supercritical CO_2_. Supercritical CO_2_ drying technology is suitable for drying aerogel materials, but due to the high price of the instrument itself and the large volume of the equipment, it is difficult to realize the common laboratory equipment.

**Scheme 1:**
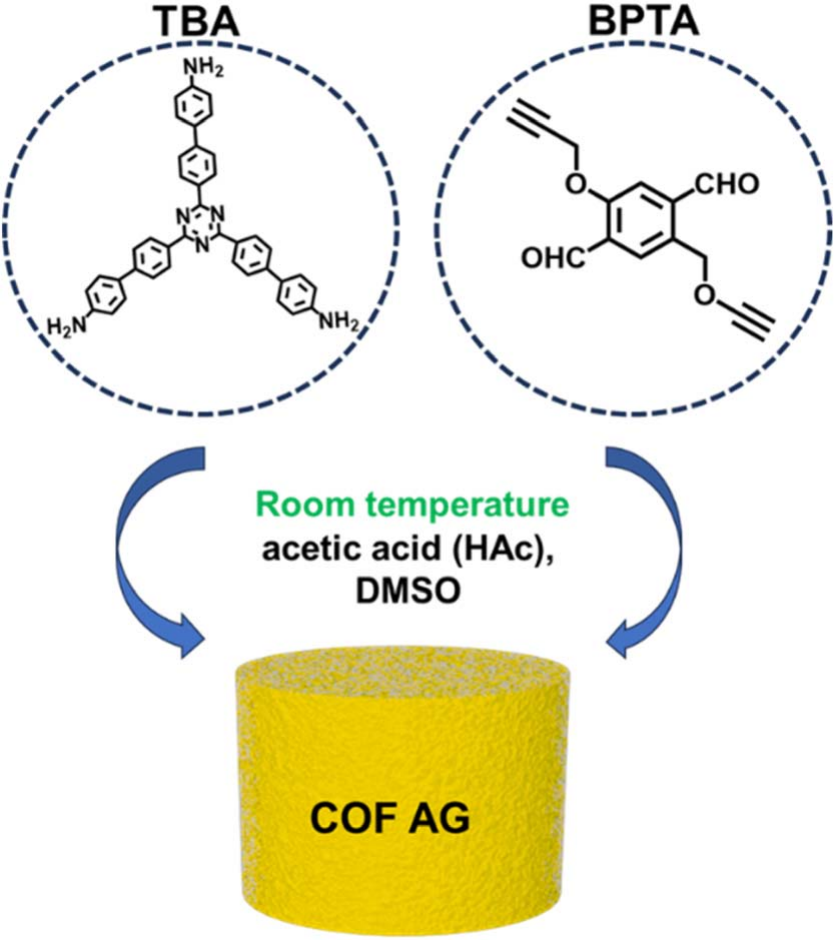
Schematic diagram of the synthesis of COF AG1. (TBA: 4′,4′′′,4′′′′′–(1,3,5-triazine-2,4,6-triyl)tris(([1,1′-biphenyl]-4-amine)); BPTA: 1,4-benzenedicarboxaldehyde, 2,5-bis(2-propyn-1-yloxy)–).

In this paper we report a simple and green method to manufacture macroscopic COF AG1 whole. COF AG1 is synthesized rapidly at room temperature, then washed and freeze-dried to prepare COF AG1 without any adhesives or additives (Scheme 1). Through the study of the transformation of amide monomer, successfully created COF AG1. Due to its strong hydrophobic property, the COF AG1 is used as an absorber in oil–water separation to remove organic pollutants, and has certain demulsification performance. Finally, the superhydrophobic COF AG1/MA sponge composites were prepared by combining hydrophobic COF AG1 materials with MA sponge at room temperature. The preparation method avoids the time-consuming reaction conditions of traditional high temperature and high-pressure reaction, and avoids the post-modification process and complex washing steps. The superhydrophobic sponge composite material is quickly prepared by vacuum drying after EtOH washing. The introduction of MA sponge reduces the amount of COF monomer, improves the adsorption capacity of the material to organic solvents and oil samples, from the previous maximum adsorption weight of 37 times to more than 120 times, the demulsification capacity of O/W emulsion increased to more than 99%, and has the ability of direct separation and continuous separation of oil and water. The test results show that the superhydrophobic sponge has good stability in strong acid, strong base and high salt solution, which provides the possibility for the application of sponge in the actual environment.

## 2. Materials reagents apparatuses and preparation methods

Experimental details are provided in ESI.†

## 3. Results and discussion

### 3.1. Material characterization and hydrophobic properties

**Fig. 1:**
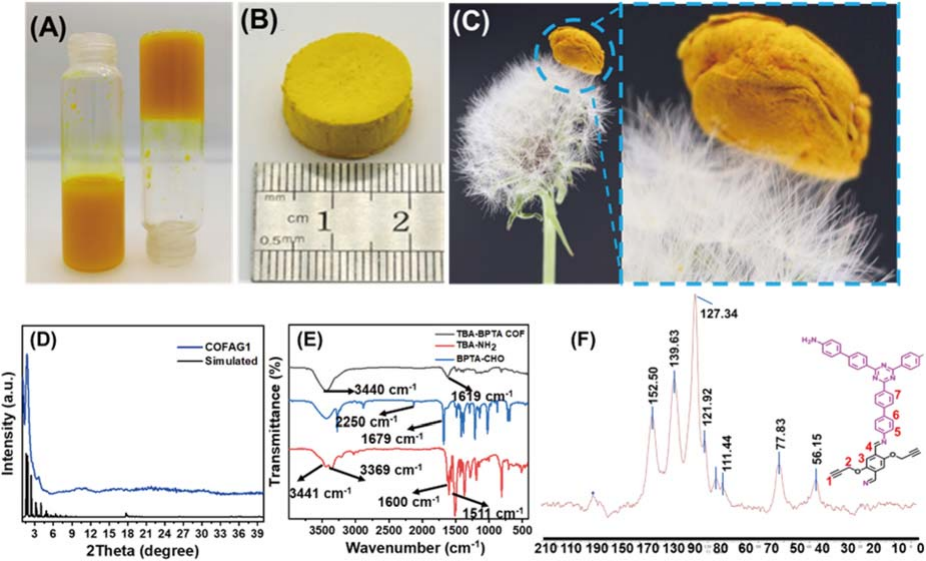
(A) COF gel photos: COF AG1; (B) COF AG1 photo; (C) photo of COF AG1 on the flower of dandelion; (D) PXRD of COF AG1; (E) FT-IR spectrum of COF AG1; (F) 13C CP-MAS NMR of COF AG1.

As showed in Fig. 1A, the COF can form uniform and stable gel structures. Then we prepared macroscopic COF AG1 through ordinary freeze-drying treatment. Fig. 1B shows a picture of regular COF AG1. Fig. 1C shows COF AG1 on dandelion flowers without crushing original structure of the dandelion flower. COF AG1 was further characterized by PXRD analysis, FTIR, N_2_ adsorption measurement, TEM and SEM. PXRD analyzed COF AG1, as shown in Fig. 1D, COF AG1 showed excellent crystallinity, and the peak position was consistent with the peak position simulated by the software, and was consistent with the peak position of the original COF powder. FT-IR characterization was performed for COF AG1, as shown in Fig. 1E, the characteristic peaks of the COF AG1 could be observed in 1619 cm^−1^, 1618 cm^−1^ and 1619 cm^−1^, respectively, which were assigned to the stretching vibrations of C=N. The aldehyde base band at 1679 cm^−1^ disappeared obviously, indicating that COF AG1 was formed through the connection of imine bonds. The peaks at 3440 cm^−1^, 3440 cm^−1^, and 3437 cm^−1^ were attributed to the stretching vibration and asymmetric bending of –OH, which may be due to the residue of acetic acid aqueous solution added in the synthesis process.^19^ To further confirm the nature of the interlayer connections in the COF AG1 structure, we carefully analyzed the 13C CP-MAS NMR spectra of the COF AG1 species. As shown in Fig. 1F, the results are consistent with the connection method we envisioned, indicating that the types of COF AG1 is connected according to the set method to generate imine COF AG1.

**Fig. 2:**
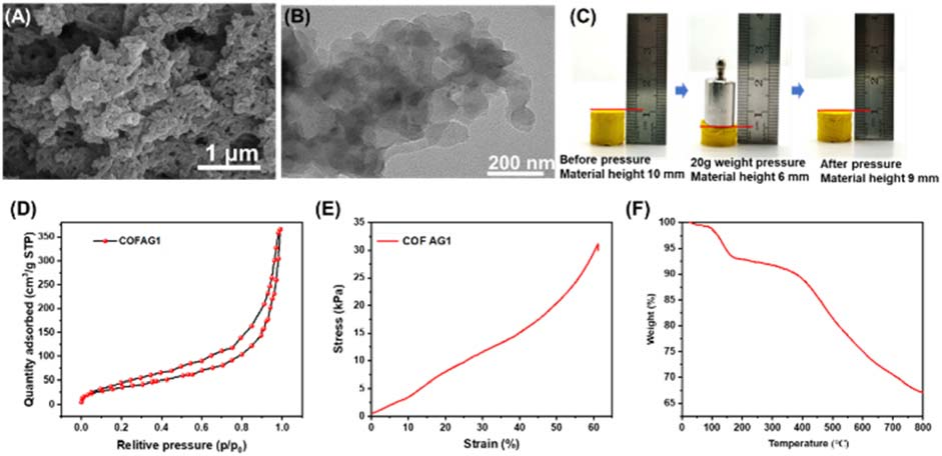
(A) SEM images of COF AG1; (B) TEM images of COF AG1. (C) Stress test of COF AG1. (D) N_2_-adsorption desorption isotherm curve of COF AG1. (E) Stress–strain curve of COF AG1. (F) Thermogravimetric analysis of COF AG1.

The material was characterized by SEM, as shown in Fig. 2A. It can be clearly observed from the SEM images that the COF AG1 was composed of strip structures and COF AG1 show many pore structures. For the synthetic COF AG1, the strip structure consists of aggregated particles and many irregular pores formed by irregular stacking of the strip structure. By comparing the images with other COF AG materials,^20,21^ it was found that the morphology of the COF AG1 materials was similar the literature, indicating the successful formation of COF AG1 materials. Then the material was characterized by TEM, as shown in Fig. 2B. The internal connection structures of the COF AG1 can also be clearly observed from the TEM images. In the images of COF AG1, it can be seen that they are mainly connected by strip structures and form aerogel structures. The TEM images of the COF AG1 materials was similar those mentioned in ref. 13, and was consistent with the results obtained from SEM images, which again proved the successful synthesis of COF AG1.

Another advantage of the COF AG1 material is its excellent compressive elasticity and flexibility. We select COF AG1 as the compression test sample. Fig. 2C shows the compression process of COF AG1 with 20 g standard weight. It can be seen that before compression, the height of COF AG1 itself is 10 mm, and when 20 g pressure is applied, COF AG1 is compressed to 5 mm. Finally, the removed weight COF AG1 was successfully restored to the height of 9 mm, indicating that the prepared COF AG1 had excellent compression elasticity and flexibility.

The porous structures of the COF AG1 was analyzed by N_2_ adsorption tests. As shown in Fig. 2D, it is found that the BET surface areas of COF AG1 is 151 m^2^ g^−1^. Compared with other relevant literature, it is found that the specific surface area of COF AG material is smaller. The possible reasons are: first, freeze-drying treatment causes irreversible shrinkage of the microporous and mesoporous structures of the original gel, which eventually leads to the collapse of relevant structures.^22,23^ Secondly, it may also be due to the use of 3% *tert*-butyl alcohol as the solvent for freeze-drying, resulting in the production of large ice crystals during the freezing process, making the contribution of large pores to the surface area of the gel insignificant.^17^

In order to further quantify the mechanical properties of the materials, we analyzed the deformation process of COF AG1 through compression to obtain the stress–strain curve of the compression process. As shown in Fig. 2E, compressive stresses of COF AG1 aerogel reaches 32 KPa at a strain rate of 60%. After compressive stress, COFs AG1 can still return to the initial state without fracture, which is mainly due to the existence of covalent bonds in the COF. That is, stronger interlayer forces during π stacking of TBA components promote the formation of more regular and rigid crystals, which are maintained by continuous growth and self-assembly.^22^ The thermal stability of the COF AG1 was investigated through thermogravimetric analysis. Show in Fig. 2F, it can be seen that the weight loss of the COF AG1 before 150 °C is about 5%, which is mainly due to the loss of water and organic solvent. COF AG1 only experiences significant weight loss around 400 °C, with a loss of approximately 15%. Prove that the COF AG1 prepared have good thermal stability.

**Fig. 3:**
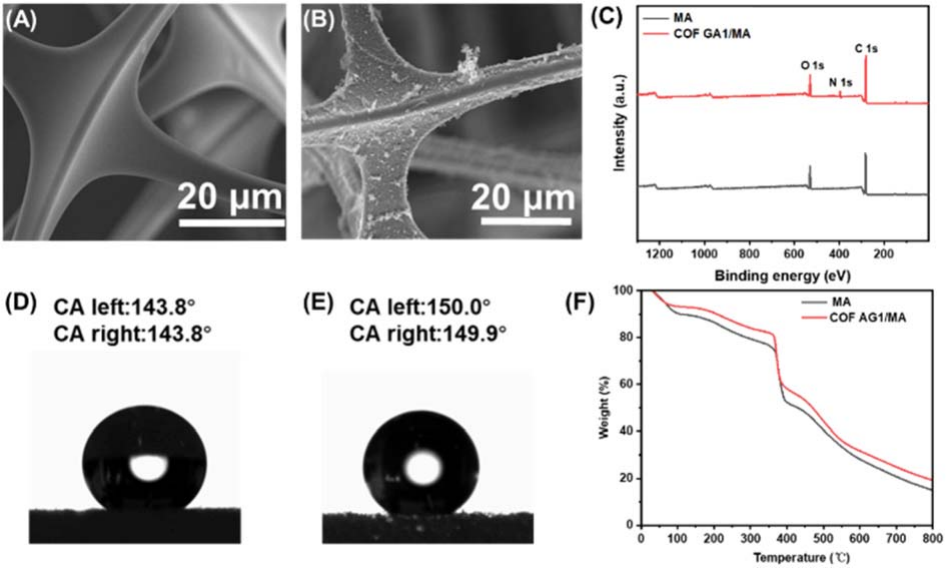
SEM image of MA sponge (A) and COF AG1/MA sponge composite (B). (C) XPS of COF AG1 and COF AG1/MA sponge composites. (D) Water Contact Angle (WCA) test of COF AG1; (E) WCA test of COF AG1/MA sponge composites; (F) thermogravimetric analysis of COF AG1 and COF AG/MA sponge composites.

In order to observe the growth of COF AG on the surface of sponges more directly, the morphology of MA sponge and COF AG1/MA sponge composites were studied by SEM. MA sponge presents three-dimensional porous structure, smooth surface after cleaning, see in Fig. 3A. The three-dimensional pore structure of the sponge can accommodate the molecules it absorbs, giving the sponge excellent adsorption capacity. The AG amino monomers of COF formed cross-linked structures and were fixed on the surface of MA sponge through the reaction of Schiff base,^23^ as shown in Fig. 3B. Instead of plugging the pores, the COF AG1 evenly envelops the three-dimensional network of sponges, forming many protruding structures.^24^ These folds make the sponge surface less smooth and provide important microstructure for enhanced hydrophobicity.

The elemental composition of the surface of COF AG1/MA sponge composite material was further characterized by XPS. From Fig. 3C, after loading COF AG1 on MA sponge, carbon content increases, and nitrogen content can only be observed on the surface of COF AG1/MA sponge composite, which may be due to the existence of triazine groups in the amino monomer of COF AG1 material. The test results shown in Fig. 3D and E demonstrate that COF AG1 and COF AG1/MA sponge composite materials exhibit strong hydrophobic properties, with a Water Contact Angle (WCA) of 143.8° for COF AG1 and 150.0° for COF AG1/MA sponge composite materials. In addition, the thermal stability of MA sponge loaded with COF AG1 material was evaluated through thermogravimetric analysis show in Fig. 3F. Compared with the individual MA sponge, the thermal stability of the MA sponge was not changed after COF AG1 composite MA. The thermal stability of the prepared COF AG1/MA sponge composite material reached around 400 °C, which is related to the good thermal stability of both the MA sponge and COF AG1 themselves. The above results indicate that COF AG1 was successfully loaded on MA sponge, demonstrating the successful synthesis of superhydrophobic COF AG1/MA sponge composite material.

### 3.2. Screening of reaction conditions

By changing the amount of solvent, catalyst, solvent and drying method, we further select the optimal synthesis conditions. Firstly, the solvent screening conditions were conducted, as shown in Fig. S1.† It was found that the use of DMSO and DMF was very important for the formation of COF gel, and uniform gel could be generated within 2 h. The only difference was that DMF was formed in a bottom-up growth mode, while DMSO was more uniform. Other solvents were also tested, including EtOH, MeOH, ACN, and HAc, but the same two monomers produced powder samples rather than uniform gels in these solvents. It has been reported in relevant literature^13^ that DMSO may participate in the formation of gel structure, so the best solvent selected in this paper is DMSO.

**Fig. 4:**
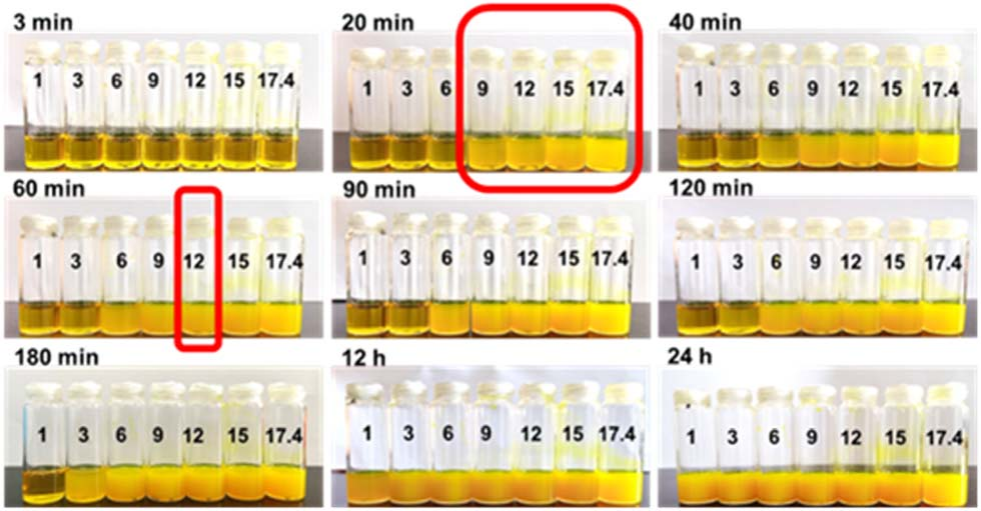
Optimization results of acetic acid concentration of COFs gel.

It has been reported that the use of HAc may also be involved in the formation of gel structures.^25^ Therefore, the concentration of HAc was optimized, as shown in Fig. 4, and it can be clearly observed that the solubility of HAc is closely related to the formation time of COF gel. The general rule observed is that when a certain volume of HAc is added, the larger the solubility of HAc is, the faster the gel will form, and the smaller the concentration of HAc, the longer the reaction time will be required. However, after 24 h, all solubility can catalyze COF to form uniform gel. Therefore, the optimal HAc concentration is 12 M.

Then, by changing the amount of solvent DMSO, the optimal concentration ratio of the reaction was screened out, as shown in Fig. S2.† With the increase of DMSO, the gel formation time also increased accordingly. The optimal amino group and aldehyde group mass of 2 mL DMSO as solvent were 11.7 mg and 7.5 mg, respectively, and the HAc was 300 μL.

In order to determine the optimum ratio of COF AG1 to MA sponge composite, we synthesized COF AG1/MA sponge composite with different proportions, taking 1 × 1 × 1 cm^3^ sponge volume as an example. We attempted to increase the load of COF AG1 for synthesis. Materials 1–5 in Fig. S3a† correspond to 1–5 mg of amino monomer and 1–5 mg of aldehyde monomer, among which the dosage of DMSO must be 5 mL. Then we dried the above synthetic materials and tested the WCA of the materials synthesized in five dosages. The results are shown in Fig. S3b.† The WCA of sponge prepared under No. 4 synthesis condition reaches 149.8°, which is not much different from that of No. 5 material. From the economic point of view, we finally choose No. 4 synthesis condition as the best preparation condition.

### 3.3. Stability of COF AG1/MA sponge composites

**Fig. 5:**
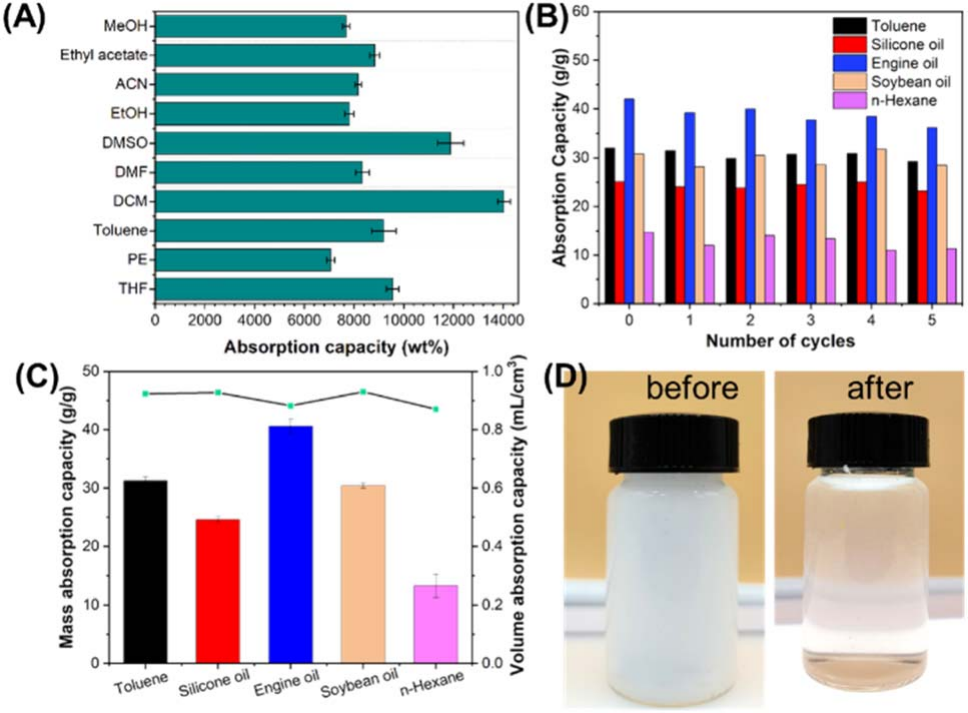
(A) Adsorption performance of COF AG1 for organic solvent; (B) the adsorption test results and cycling test of COF AG1 on actual oil samples. (C) Mass and volume adsorption capacity of COF AG1 on actual oil samples. (D) Demulsifying effect of COF AG1.

COF AG1 further tested the adsorption capacity of different organic solvents. Ten common organic reagents were selected (MeOH, EAC, CAN, EtOH, DMSO, DMF, DCM, TL, PE, THF). Fig. 5A shows that COF AG1 have rich macroscopic pores and microscopic structures. The adsorption capacity of COF AG1 for various organic solvents is very high, up to 36 times its own weight, indicating that the porous network of COF AG1 helps to fix solvent molecules and promote adsorption performance. In addition, the high adsorption capacity of organic solvents is also related to the density of the solvent itself. Compared with relevant literature, the adsorption capacity of COFA-1 on various organic solvents reported by Li *et al.*^20^ is up to 27 times of its own weight, while the maximum adsorption capacity of TTAP-OMePDA aerogel introduced by Zhu *et al.*^19^ is up to 35 times of its own weight. Zhang *et al.*^23^ reported that the maximum adsorption capacity of COF foam on organic solvent was 5.5 times more than its own weight. COF AG1 conducted adsorption tests on samples of toluene, silicone oil, vacuum pump oil, edible soybean oil, and *n*-hexane, as shown in Fig. 5B. It can be seen that except for silicone oil and *n*-hexane, COF AG1 has an adsorption capacity of more than 30 times its own weight for the other three samples, especially for the vacuum pump oil sample, which has an adsorption capacity of more than 40 times its own weight. The recyclability and reusability of COF AG1 were further tested. After adsorbing the oil sample, it was subjected to solvent exchange with EtOH until the EtOH solution became clear, and then vacuum dried at 60 °C before reuse. After 5 cycles, COF AG1 still maintains high adsorption capacity for five oil samples, indicating that the prepared COF AG1 has good reusability. COF AG1 tested the mass adsorption capacity and volume adsorption capacity of five actual oil samples. Shown in Fig. 5C, it can be observed that the mass adsorption capacity varies due to the different densities and viscosities of each oil sample. However, from the volumetric adsorption capacity, it can be seen that COF AG1 has an adsorption capacity higher than 0.8 mL cm^−3^ for each oil sample, indicating that COF AG1 has good adsorption capacity for all five oil samples.

As we all know, oil–water lotion is easy to form after oil–water mixing, and oil–water lotion can be divided into water in oil (W/O) lotion and oil in water (O/W) lotion. W/O lotion is easy to separate without demulsification. The separation of O/W type lotion is relatively complicated, because oil droplets are dispersed in water in the form of small droplets and wrapped by water, and the oil–water separation can be smoothly achieved only after demulsification treatment.^26^ The prepared COF AG1 was tried for demulsification. The Fig. 5D shown in the photo images before and after demulsification using COF AG1 (the picture on the left shows before demulsification, and the picture on the right shows after demulsification). It can be observed that the prepared emulsion is milky white after uniform demulsification. After adsorption by COF AG1, the milky white becomes pale and the solution becomes transparent. The results show that COF AG1 has a certain demulsification ability due to its superhydrophobic property.^27,28^

## 4 Conclusions

In summary, we have developed a simple and green method for preparing macroscopic COF gels and AG1 monoliths. COF gel was rapidly synthesized at room temperature, washed and freeze-dried to prepare COF AG1 without any adhesives or additives. The COF AG1 exhibited exceptional hydrophobicity, thermal, and chemical stability. The ordered channels and superhydrophobic void environments of the COF AG1 effectively prevent the permeation of water molecules during oil transport. This unique property allows the COF AG1 to demonstrate exceptional separation performance for oil/water mixtures and water-in-oil emulsions. Remarkably, the separation efficiencies and oil fluxes remain consistent even after multiple separation cycles. The porous structure and hydrophobic property makes COF AG1 as promising absorbent for oil/water separation with high adsorption capacity and good reusability.

## Data availability

All data that support the findings of this study are included within the article.

## Author contributions

LBL: conceptualization, methodology, investigation, formal analysis, writing – original draft preparation; QMD: conceptualization, data curation, investigation, validation, writing – original draft preparation; LSY: supervision, validation; FT: supervision, validation; RC: supervision, validation; ZX: supervision, validation; HZ: conceptualization, supervision, and validation; CBL: conceptualization, project administration, supervision, validation, funding acquisition.

## Conflicts of interest

The authors declare that they have no known competing financial interests or personal relationships that could have appeared to influence the work reported in this paper.

## Supplementary Material

RA-015-D4RA08201J-s001
