# Supplementary material for: Hydrophobic covalent organic frameworks: a green synthesis approach for efficient oil/water separation
Source: RSC Adv. 2025 Feb 13;15(6):4768–73. doi: 10.1039/d4ra08201j (PMC11822353; doi:10.1039/d4ra08201j)
Supplement: RA-015-D4RA08201J-s001 [file RA-015-D4RA08201J-s001.pdf]

Supplementary Information (SI) for RSC Advances.
This journal is © The Royal Society of Chemistry 2025

Supplementary Information

Hydrophobic covalent organic frameworks: A green synthesis approach for efficient oil/water separation

Bilian Li,[a] Quanmei Duan,[a] Lishen Yang,[a] Tan Feng,[a] Chang Ru,[c] Xin Zhang,[c] Hui Zhao,*[b] Can-Peng Li*[a]

[a]School of Chemical Science and Technology, Yunnan University, 2 North Cuihu Road, Kunming 650091, People's Republic of China

[b]Laboratory for Conservation and Utilization of Bioresource, Yunnan University, 2 North Cuihu Road, Kunming 650091, People's Republic of China

[c]Research Center for Safety and Environment (Double Carbon Research Center), PipeChina Institute of Science and Technology, Tianjin 300457, China

Bilian Li and Quanmei Duan contributed equally to this work.

Corresponding Authors：Hui Zhao, Can-Peng Li

E-mail: lcppp1974@sina.com (C.-P. Li); zhaohui@ynu.edu.cn (H. Zhao)

Supplementary Methods

1.1. Materials and reagents

1,3,5-tri (4-aminophenyl) benzene (TAPB); 2,5-bis (2-propary-1-methoxy) -1, 4-phenyldiformaldehyde (BPTA); 4,4-(1,3, 5-triazine-2,4, 6-triyl) tri ([1, 1-biphenyl] -4-amine) (TBA); 5 '- (4' - amino [1, 1 '- al phenyl) - 4 - base) [1, 1', 4 ', 1 ', 3 ', '1', 4 ', ' '1' '''- five biphenyls] - 4, 4' ''' - diamine (FBDA) derived from Ji Lin Zhong scientific research and technology co., LTD. Acetonitrile (ACN), anhydrous ethanol (EtOH), tetrahydrofuran (THF), toluene, N,N-dimethylformamide (DMF), methanol (MeOH), dimethyl sulfoxide (DMSO), petroleum ether (PE), ethyl acetate, dichloromethane (DCM), trichloromethane, n-hexane, tert-butanol, methylene Blue, Sudan III, N-hexadecane, sodium dodecyl sulfate, were supplied by Adamas-beta Reagent Co., LTD. (Shanghai, China). Silicone oil and pump oil were supplied by Shanghai Titan Technology Co., LTD. Soybean oil was supplied by Yi hai Food Co., LTD. Whole Milk was supplied by Dali Lesier Dairy. Melamine (MA) sponge was supplied by Huizhou Best Sheng Technology Co., LTD. In this work, all of the above reagents were analytically grade and required no further purification for use. All aqueous solutions were prepared with deionized water (DW, 18 MΩ cm^{-1}).

1.2. Apparatus

The morphologies of the prepared samples were characterized by transmission electron microscopy (TEM; JEM 2100, Japan). X-ray photoelectron spectroscopy (XPS) measurements were performed with Al Kα X-ray radiation as the X-ray source for excitation, on an ESCALAB-MKII spectrometer (VG Co., United Kingdom). Contact Angle tester was used for contact Angle test (Kunshan Beidou Precision Instrument Co., LTD.) Testing materials using solid-state nuclear magnetic resonance

spectrometer (Bruker, Switzerland). Stress-strain curves of materials tested using universal mechanical testing machine (Mecmesin UK). Using a rotating X-ray polycrystalline diffractometer (Nishiko Corporation). Use freeze dryer (Beijing Yibokang Company). Deionized water was prepared by Milli-Q water purification system (Millipore, USA).

1.3. Preparation of COF AG1

To prepare TBA-BPTA COF AG1 (COF AG1), take a clean beaker and add 11.7 mg TBA, 7.25 mg BPTA and 5 mL DMSO into the beaker for ultrasound for 3 min until the solution is clarified. In the stirring state, 150 μL glacial acetic acid with a concentration of 6 mol/L was added drop by drop, and the rotating speed was 500 rpm to continue magnetic stirring for 1 min. The mouth of the beaker was sealed with a sealing film and left to react at room temperature for 12 h. After the reaction was complete, THF and EtOH were used for solvent exchange three times, and then tert-butanol with water content of 3% was used for solvent exchange three times. COF AG1 material was freeze-dried.

1.4. Preparation of superhydrophobic COF AG1/MA sponge composites

In order to prepare TBA-BPTA COF AG1/MA sponge composite, 11.7 mg TBA, 7.5 mg BPTA and 9 mL DMSO were added into a clean beaker, and the solution was ultrasonic until it was clarified. Finally, 6 mol/L, 300 μL acetic acid solution was added and stirred for 3 min. After mixing well, add a clean MA sponge with a volume of 8 cm3 to make the liquid evenly immersed in the whole sponge. Seal the mouth of the beaker with a sealing film and let it react at room temperature for 6 h. After the reaction was complete, the excess solvent in the upper layer was removed, and the sponge was washed with EtOH three times to drain the excess EtOH. Superhydrophobic TBA-BPTA COF AG1/MA (COF

AG1/MA) sponge composite was obtained by placing the sponge in a vacuum drying oven at 60 ^{o}C for 12 h.

1.5. Preparation of oil-in-water emulsion

According to the literature method [1], 24 mL sodium dodecyl sulfate aqueous solution with a concentration of 0.5 mg/mL was prepared first, then 1mL toluene was added into the solution, and the mixture was homogenized for 1 h at the speed of 1500 r/min by high-speed magnetic stirring, to obtain oil-in water emulsion.

1.6. Pretreatment of melamine (MA) sponge

The MA sponge block was taken and cut into a cube with a length of 2 cm wide and 2 cm high and a volume of 8 cm^3. The sponge was soaked with EtOH for 2 hours to remove impurities. Finally, the sponge was washed with DW for three times to remove residual EtOH. Dry in oven at 60 ^{o}C under atmospheric pressure for 12 h to get clean MA sponge.

1.7. Adsorption capacity and separation performance

Adsorption capacity and separation performance In practice, absorptive capacity is an important factor in the evaluation of absorbent materials. The adsorption capacity of three kinds of COF AG1 was tested by typical adsorption tests.

Specifically, the weight of the clean COF AG1 is first recorded as W_0 (mg). The clean weighed COF AG1 sample was then placed in oil or organic solvent until adsorption saturation. The material was re-weighed with a recorded weight of W_1 (mg). Finally, the following formula (1) was used to calculate the adsorption capacity, where Q m/m (wt%) represents the adsorption capacity of COF AG1 [2].

$$Q_{m/m} = \frac{W_1 - W_0}{W_0} \times 100\% \quad (1)$$

The volume absorption capacity (Q v/v) is calculated by the formula (2) as follows:

$$Q_{v/v} = \frac{W_1 - W_0}{W_0} \times \frac{d_s}{d_l} \quad (2)$$

ds and dl are the bulk density of the absorber and the density of the absorbed liquid, respectively [3].

The separation emulsion performance was tested with COF AG1. The prepared 20 mg COF AG1 was placed in 25 mL oil-in-water emulsion and oscillated for 1 min until the emulsion became transparent. Optical microscope images of oil droplets in emulsion were measured by digital microscope. In order to further study the separation effect of COF AG1 on emulsion, the separation efficiency (R) of emulsion was calculated by equation (3), in which C0 (mg/L) and C1 (mg/L) are toluene content before and after separation, respectively [4].

$$R = \frac{C_0 - C_1}{C_0} \times 100\% \quad (3)$$

To assess the oil-water separation performance of preparing superhydrophobic COF AG1/MA sponge composites, 20 mL of organic solvent or oil was mixed with 20 mL of water and the mixture

was poured into a beaker and the organic solvent or oil was extracted using a vacuum pump. The weight of water before and after separation was recorded, and the separation efficiency was calculated using equation (4). η (%) represents the separation efficiency of the immiscible oil and water mixture, and m0 (g) and m_1 (g) represent the weight before and after separation, respectively.

$$\eta = \frac{m_1 - m_0}{m_0} \times 100\% \qquad (4)$$

Supplementary Figures and Tables

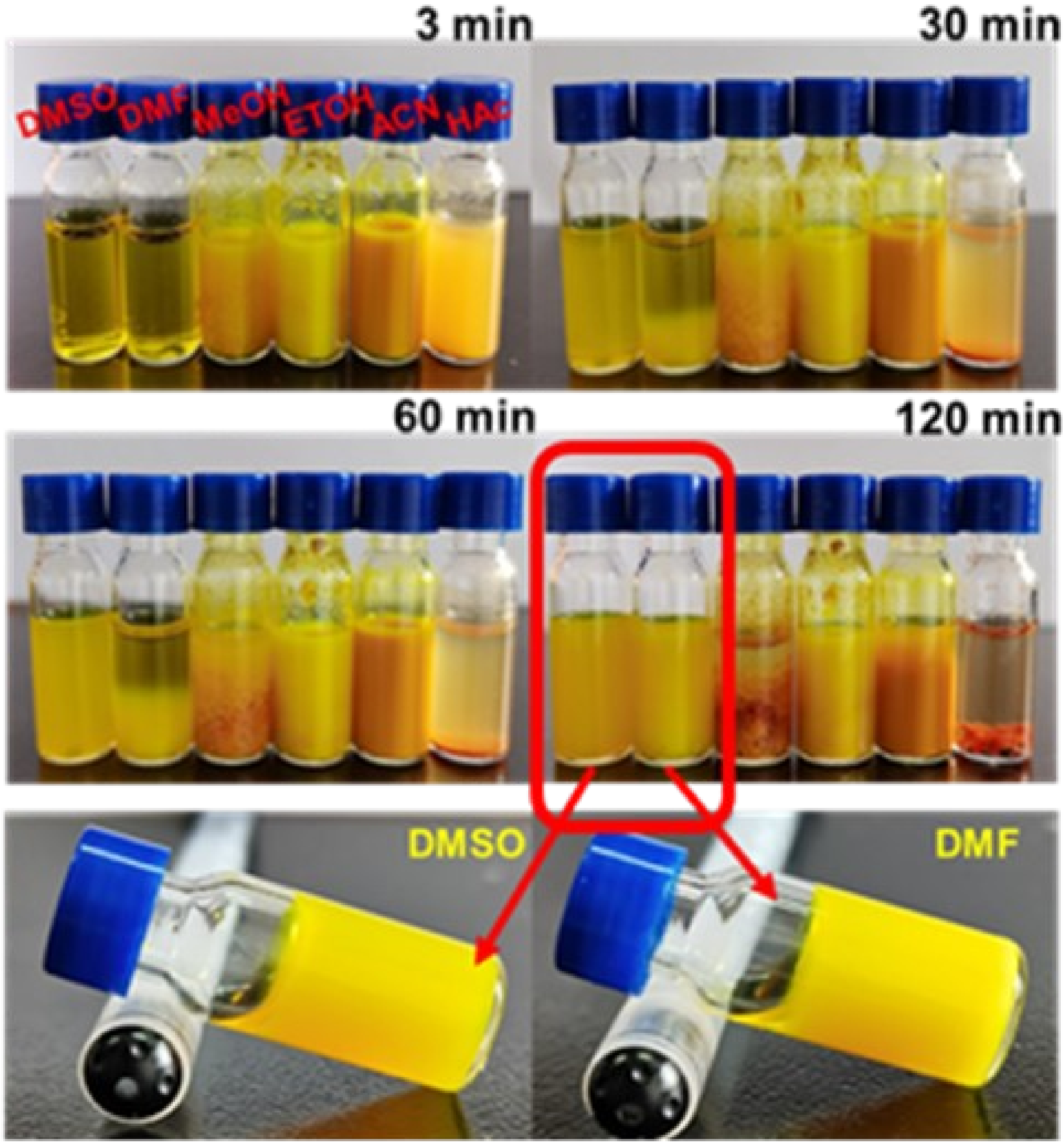

Figure. S1. Solvent optimization of COF gels.

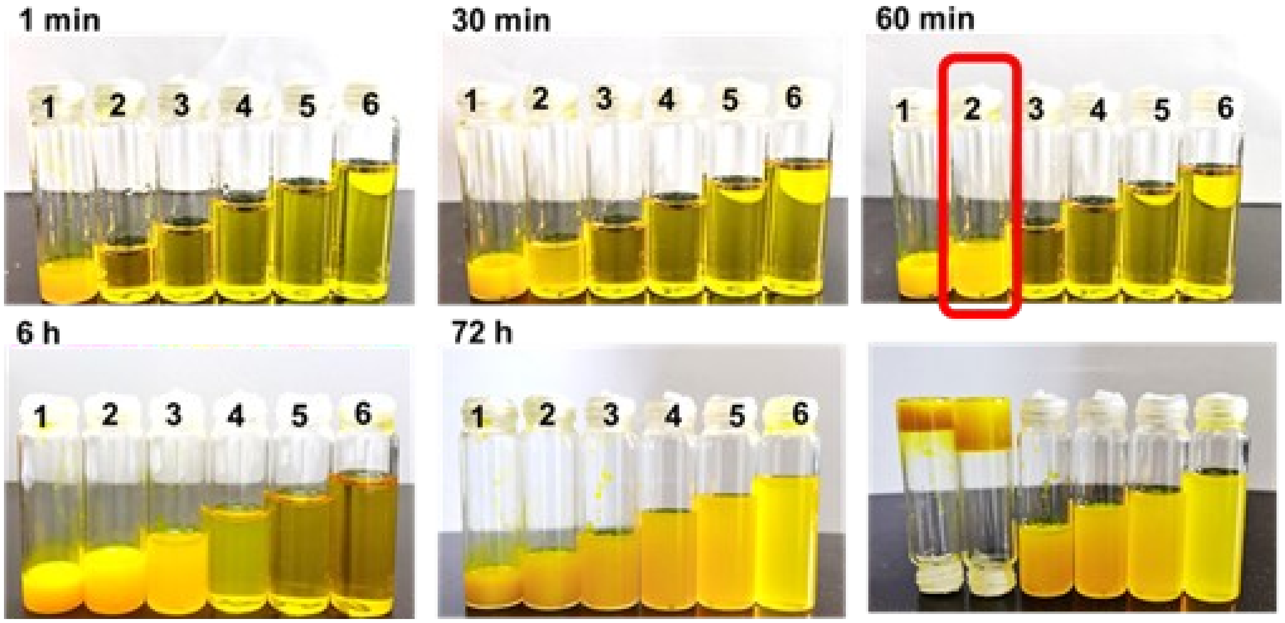

Figure. S2. DMSO concentration optimization results of COFs gel.

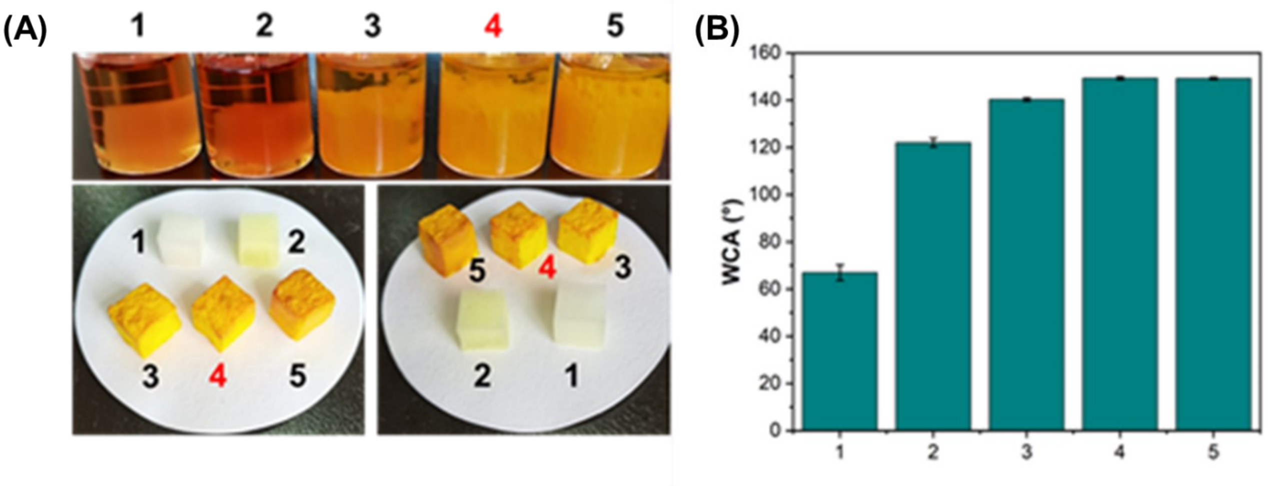

Figure. S3. (**A**)Synthesis process of COF AG1 and MA sponge with different dosage. (**B**) Contact Angle of composite material synthesized by COF AG1 and MA sponge with different dosage.

References

1. Deryło-Marczewska A, Chrzanowska A, Marczewski A W. Morphological, structural and physicochemical characteristics of the surface of mesocellular silica foam with the adsorbed OVA and BSA proteins [J]. Microporous and Mesoporous Materials, 2020, 293: 109769.

2. Zhu D, Zhu Y, Yan Q, et al. Pure crystalline covalent organic framework aerogels [J]. Chemistry of Materials, 2021, 33(11): 4216-4224.

3. Ge J, Wang F, Yin X, et al. Polybenzoxazine-functionalized melamine sponges with enhanced selective capillarity for efficient oil spill cleanup [J]. ACS Applied Materials and Interfaces, 2018, 10(46): 40274-40285.

4. Pang Y, Yu Z, Chen H, et al. Superhydrophobic polyurethane sponge based on sepiolite for efficient oil/water separation [J]. Journal of Hazardous Materials, 2022, 434: 128833.
